# The Ionic Product of Water in the Eye of the Quantum Cluster Equilibrium

**DOI:** 10.3390/molecules27041286

**Published:** 2022-02-14

**Authors:** Barbara Kirchner, Johannes Ingenmey, Michael von Domaros, Eva Perlt

**Affiliations:** 1 Mulliken Center for Theoretical Chemistry, Institute for Physical and Theoretical Chemistry, Beringstr. 4, 53115 Bonn, Germany; 2 CNRS, Physico-Chimie des Électrolytes et Nanosystèmes Interfaciaux, Sorbonne Université, F-75005 Paris, France; johannes.ingenmey@sorbonne-universite.fr; 3 Department of Chemistry, Philipps-Universität Marburg, Hans-Meerwein-Straße 4, 35032 Marburg, Germany; mvondomaros@uni-marburg.de; 4 Otto Schott Institute of Materials Research, Faculty of Physics and Astronomy, Friedrich-Schiller-Universität Jena, Löbdergraben 32, 07743 Jena, Germany; eva.von.domaros@uni-jena.de

**Keywords:** statistical thermodynamics, water, ionic product, vaporization, quantum cluster equilibrium, density functional theory

## Abstract

The theoretical description of water properties continues to be a challenge. Using quantum cluster equilibrium (QCE) theory, we combine state-of-the-art quantum chemistry and statistical thermodynamic methods with the almost historical Clausius–Clapeyron relation to study water self-dissociation and the thermodynamics of vaporization. We pay particular attention to the treatment of internal rotations and their impact on the investigated properties by employing the modified rigid-rotor–harmonic-oscillator (mRRHO) approach. We also study a novel QCE parameter-optimization procedure. Both the ionic product and the vaporization enthalpy yield an astonishing agreement with experimental reference data. A significant influence of the mRRHO approach is observed for cluster populations and, consequently, for the ionic product. Thermodynamic properties are less affected by the treatment of these low-frequency modes.

## 1. Introduction

Since the dawn of electrochemistry at the end of the 18th century, scientists have sought to understand the decomposition of water due to an electric current. In 1805, Theodor Grotthuss developed the first theory to describe this phenomenon [1,2,3], in which he assumed that water molecules become polarized under the influence of electric voltages of electrodes, that is, oxygen atoms become negative and hydrogen atoms become positive. The attraction and repulsion of the electrodes consequently lead to an alignment of the molecules, creating water wires that span the electric cell. Grotthuss further assumed that if the electromotive force is strong enough, the outer hydrogen and oxygen atoms of the wire, those in contact with the electrodes, will separate from their corresponding molecules, forming molecular oxygen and hydrogen at the respective electrode. The residual atoms subsequently recombine along the water wire, organizing as a new set of molecules. About 50 years later, Rudolf Clausius recognized that water molecules can also dissociate spontaneously without an applied electric field; the applied voltage just regulates and accelerates the movement of charged particles toward the corresponding electrode [4]. This self-ionization, measured in terms of pH and characterized by the ionic product, $K_{w}$ (or $pK_{w}=-\mathrm{lg}K_{w}$), is now a cornerstone of our understanding of aqueous acid–base chemistry.

Grotthuss’s breakthrough ideas of polarization as well as continuous dissociation and reformation of water molecules were embraced by Danneel in 1905 to explain the unusually high mobility of protons in liquid water [5], a process which is nowadays referred to as the Grotthuss diffusion mechanism and has far-reaching consequences in biological systems (see Ref. [6] for a comprehensive special issue on the subject).

In technical applications, on the other hand, pH is an important stimulus. The most basic examples are probably pH indicators, whose electronic properties—and thereby their color—are altered by proton activity. More advanced examples include pH-responsive polymers, which have far-reaching applications in catalysis [7], nanomedicine [8], materials science [9], and many other fields [10,11].

Today, more than 200 years after the discoveries of Grotthuss and Clausius, theoretical chemists and physicists have an arsenal of advanced techniques to study proton transfer and transport in liquid water at high levels of theory through static calculations and dynamic simulations [12,13,14,15,16,17,18,19]. Yet, the actual computation of the ionic product is still a cumbersome task. The major challenges lie in the corresponding ionic concentrations being too small to allow direct simulations as well as water being extremely efficient in catalyzing its own dissociation, complicating static calculations. The latter can be demonstrated beautifully by comparing $pK_{w}=14$ at standard state conditions against the value obtained for the hypothetical gas-phase dissociation of a water molecule, $pK_{w}^{\left(g\right)}=285$, at the B3LYP/6-311++G** level of theory [20]. In ab initio molecular dynamics simulations, the challenges are typically overcome through the application of artificial biases or constraints and vast amounts of computational power [21,22]. Static quantum-chemical calculations often resort to combinations with continuum theories and classical models [23,24,25,26,27,28], tainting the first principles character of the calculation.

In 2022, we remember Grotthuss’s 200th deathday and the bicentenary of Rudolf Clausius’s birth. In 2021, we also celebrated the 80th birthday of Frank Weinhold, to whom this article is dedicated. It turns out that Weinhold’s quantum cluster equilibrium (QCE) theory [29,30] establishes two important links to the works of Grotthuss and Clausius. QCE is a theory to describe associated fluids and their mixtures through representative cluster structures. The cluster weights are related to the chemical potential of the clusters, taking vibrational and rotational entropic contributions through application of the rigid-rotor–harmonic-oscillator (RRHO) approximation into account. A hallmark of QCE theory is the ability to treat phase transitions, such as in Weinhold’s seminal work, where he employed the Clausius–Clapeyron relation to describe the thermodynamics of water vaporization [30]. It turns out that QCE also provides alternative means to studying aqueous dissociation processes, void of the complications mentioned above. In the past, QCE has thus been successfully applied to compute the ionic product of water and dissociation constants of other weak acids as a function of temperature [20,31,32].

In this contribution, we build upon these foundations. Inspired by the historical background and driven by the outstanding performance of the QCE approach in describing the ionic product of water, we reconsider both the ionic product and the Clausius–Clapeyron relation for vaporization, using the so-called modified rigid-rotor–harmonic-oscillator (mRRHO) [33] model. Therein, those vibrational modes of the cluster structures, that actually represent internal rotations of molecular units and that occur at small wavenumbers, are appropriately treated by the partition function of a hindered rotor, rather than a harmonic oscillator.

The article is structured as follows. In Section 2, we revisit essential elements of QCE theory and the computational protocol, followed by the presentation and discussion of the results in Section 3. Conclusions are provided in Section 4.

## 2. Methods

### 2.1. QCE Theory

Quantum cluster equilibrium theory treats the coupled chemical equilibria between a monomer structure, $C_{1}$, and a set of clusters, $\left\{C_{℘}|℘=2,\ldots ,N\right\}$, built therefrom, according to

(1) $$i_{℘}C_{1}⇋C℘\;,$$

where *℘* is a running index labelling the clusters and $i_{℘}$ denotes the cluster size (number of monomers). All clusters are characterized quantum-chemically: their geometries are optimized, their ground-state energy is determined, and a vibrational frequency analysis is performed. Furthermore, cluster volumes, $v_{℘}$, are computed. QCE theory has been fully described several times in the past [29,34,35,36,37]. Here, we only review basic aspects of the theory and recent modifications [38] to the partition functions.

Assuming independent clusters with fixed populations, $\left\{N_{℘}\right\}$, and independent degrees of electronic, translational, vibrational, and rotational freedom, the canonical partition function, $Q\left(\left\{N_{℘}\right\},V,T\right)$, at constant volume, *V*, and temperature, *T*, is given by

(2) $$Q\left(\left\{N_{℘}\right\},V,T\right)=\prod_{℘=1}^{N}\frac{1}{N_{℘}!}{\left[q^{\mathrm{elec}}\left(V,T\right)q^{\mathrm{trans}}\left(V,T\right)q^{\mathrm{vib}}\left(T\right)q^{\mathrm{rot}}\left(T\right)\right]}^{N_{℘}}\;,$$

where the factor $1/N_{℘}!$ accounts for the indistinguishability of like clusters and the terms denoted by $q^{\mathrm{dof}}$ are individual cluster partition functions corresponding to the respective degree of freedom (dof).

For the electronic partition function, only the ground-state electronic energy and a mean-field term, describing average intercluster interactions, are considered:

(3) $$\varepsilon_{\mathrm{mf}}=-a_{\mathrm{mf}}i_{℘}\rho \;,$$

where $a_{\mathrm{mf}}$ is called mean-field parameter and ρ is the particle density of the system. The remaining individual partition functions are given by textbook [39] expressions for the following simple models: particle in a box (translation), harmonic oscillator (vibrations), and rigid rotor (rotations). The combination of the last two is typically referred to as rigid-rotor–harmonic-oscillator (RRHO) approximation. The accessible volume that enters into the translational partition function is reduced by an excluded volume term taking the finite size of each cluster into account:

(4) $$V^{\mathrm{excl}}=b_{\mathrm{xv}}\sum_{℘=1}^{N}N_{℘}v_{℘}\;,$$

where $b_{\mathrm{xv}}$ is called excluded volume scaling parameter and $v_{℘}$ is the cluster volume.

Although the theory is developed in the canonical ensemble, neither populations nor volume are input parameters in a QCE calculation. Instead, the volume is chosen to be consistent with a specified, external pressure,

(5) $$P=k_{B}T{\left(\frac{\partial \ln Q}{\partial V}\right)}_{\{N_{℘}\},T}\;,$$

where $k_{B}$ is Boltzmann’s constant. The populations are chosen to satisfy the condition for chemical equilibrium,

(6) $$\frac{\mu_{1}}{i_{1}}=\frac{\mu_{2}}{i_{2}}=\cdots =\frac{\mu_{N}}{i_{N}}\;,$$

where $\mu_{℘}$ are the chemical potentials of the clusters. These two constraints lead to a set of coupled polynomial equations, which can be solved by the Peacemaker QCE code [34,35,37].

As indicated above, QCE theory contains two adjustable parameters, $a_{\mathrm{mf}}$ and $b_{\mathrm{xv}}$, which are typically adjusted to fix the density at ambient conditions and the temperature of phase transition, although other options are conceivable. The special case $a_{\mathrm{mf}}=0,\;b_{\mathrm{xv}}=1$ is called QCE(0) and corresponds to the description of a cluster gas.

Instead of scanning all possible combinations of $a_{\mathrm{mf}}$ and $b_{\mathrm{xv}}$ on a coarse grid as in earlier studies, the current version of Peacemaker [34,35,37] features an implementation of the downhill simplex algorithm [40] which speeds up the calculations tremendously [41] while also increasing the accuracy of the parameter optimization. Here, $a_{\mathrm{mf}}$ and $b_{\mathrm{xv}}$ were optimized within a range of 0.0 to 2.0 J$\;m^{3}$ mol${}^{-2}$ and 0.5 to 2.0, respectively, so as to minimize the deviations of the computed density at room temperature and of the phase transition temperature from their respective experimental values. Once the parameters are optimized, the accessible temperature and pressure ranges of the QCE approach are, in principle, limited only by the condition that Boltzmann statistics apply, i.e., the number of particles *N* is much smaller than the number of accessible energy states $N_{\epsilon }$. This condition is typically fulfilled by molecular solvents at room temperature, but imposes a system-dependent lower boundary to the accessible temperature range.

Note that the computational bottleneck of a typical QCE study lies in the quantum chemical optimization of the clusters, whereas the QCE calculation itself will finish within seconds to minutes. This allows, in principle, for the inclusion of large-scale cluster sets containing hundreds of clusters [38] if the underlying quantum chemical method makes it feasible. Here, we opted for higher level quantum-chemical methods at the cost of using a smaller but representative cluster set.

### 2.2. Modified Partition Functions

In the following, we briefly review the modified rigid-rotor–harmonic-oscillator (mRRHO) QCE approach, which we have recently implemented [38] in the Peacemaker [34,35,37] QCE code. In this approach, the vibrational partition function is modified in accordance to the model presented in Ref. [33],

(7) $$\begin{array}{cc} q_{℘}^{\mathrm{vib}}\left(T\right) & =\prod_{i}{\left[q_{\mathrm{HO}}^{\mathrm{vib}}\left(T,\omega_{i}\right)\right]}^{f(\omega_{i})}\;\cdot \;{\left[q_{\mathrm{HR}}^{\mathrm{vib}}\left(T,\omega_{i}\right)\right]}^{1-f(\omega_{i})}\;, \end{array}$$

where the product is over all normal modes *i* with frequencies $\omega_{i}$, $q_{\mathrm{HO}}^{\mathrm{vib}}$ is the traditional partition function of a harmonic oscillator, and $q_{\mathrm{HR}}^{\mathrm{vib}}$ is the partition function of a hindered rotor. The latter is given by

(8) $$\begin{array}{c} q_{\mathrm{HR}}^{\mathrm{vib}}\left(T,\omega_{i}\right)=\sqrt{\frac{2\mu^{\prime }\left(\omega_{i}\right)k_{B}T}{\pi \hbar^{2}}}\;,\;\mathrm{with}\;\mu^{\prime }\left(\omega_{i}\right)=\frac{\mu \left(\omega_{i}\right){\bar{I}}_{℘}}{\mu \left(\omega_{i}\right)+{\bar{I}}_{℘}}\;\mathrm{and}\;\mu \left(\omega_{i}\right)=\frac{\hbar }{4\pi \omega_{i}}\;, \end{array}$$

where ${\bar{I}}_{℘}$ is the average moment of inertia of the cluster and $\mu \left(\omega_{i}\right)$ is the moment of inertia corresponding to the normal mode. The modified partition function corresponds to that of a regular harmonic oscillator for high-frequency modes and to a hindered rotor for low-frequency modes. The switching between the two models is handled by a switching function, $f(\omega_{i})$, for which the Chai–Head-Gordon damping function is applied [42],

(9) $$\begin{array}{cc} f(\omega_{i}) & ={\left[1+{\left(\frac{\omega_{0}}{\omega_{i}}\right)}^{4}\right]}^{-1}\;. \end{array}$$

Therein, $\omega_{0}$ is a user-defined rotor cutoff value. In this work, we applied two distinct cutoffs: 50 and 100 cm^−1^. The former is a sensitive choice in the recommended range of reasonable values between 20 and 100 cm^−1^ and the latter represents an upper limit. Choosing both allows us to investigate the influence of this parameter in a sensible way [38].

### 2.3. Systems Investigated

A set of clusters representative of the liquid phase of water was constructed. The set contains both regular and zwitterionic clusters and has been previously employed to compute the ionic product of water [20].

**Figure 1:**
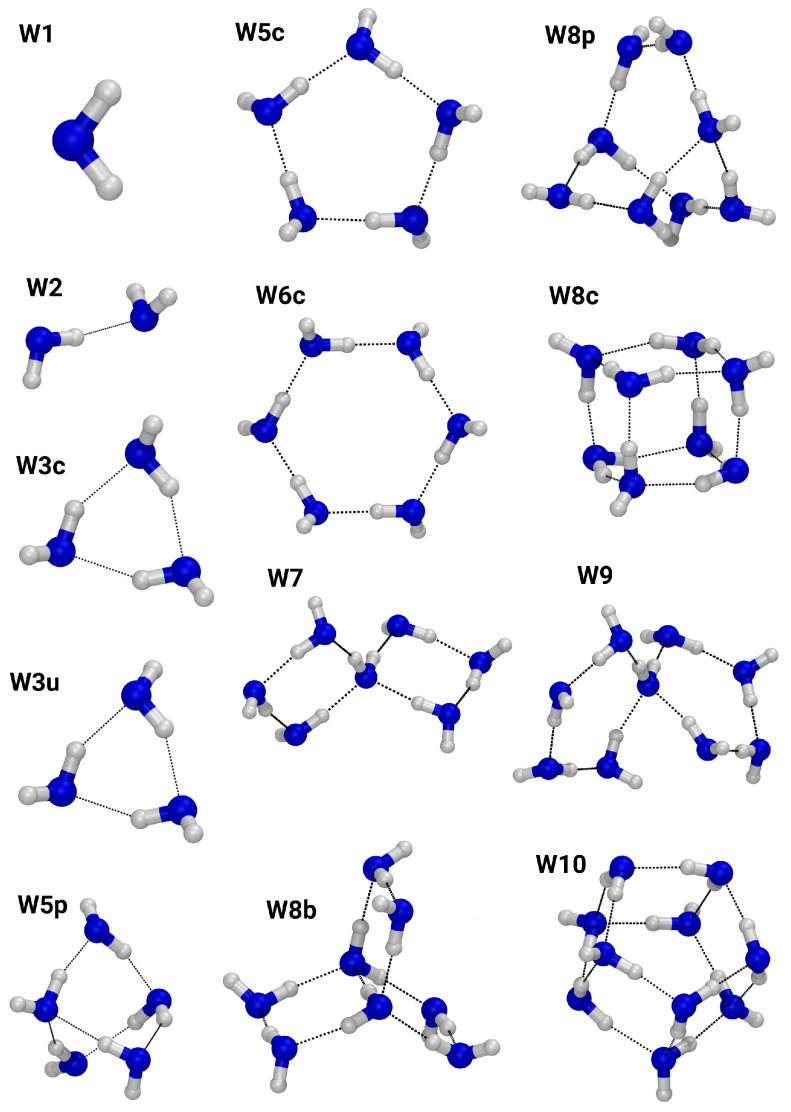
Ball-and-stick representation of the neutral water clusters. Oxygen: blue; hydrogen: white.

Figure 1 depicts the regular water clusters, with oxygen atoms colored blue and hydrogen atoms colored white. Their sizes range from the water monomer, W1, to a decamer cluster, W10. The trimers, W3c and W3u, as well as the clusters W5c and W6c exhibit exclusively two-fold hydrogen bond motifs, i.e., each water molecule accepts and donates one hydrogen bond. Three-fold hydrogen bond coordination can be found in clusters W5p, W8c, W8p, and W10. A four-fold hydrogen bond pattern is present in the clusters W7, W8b, and W9.

**Figure 2:**
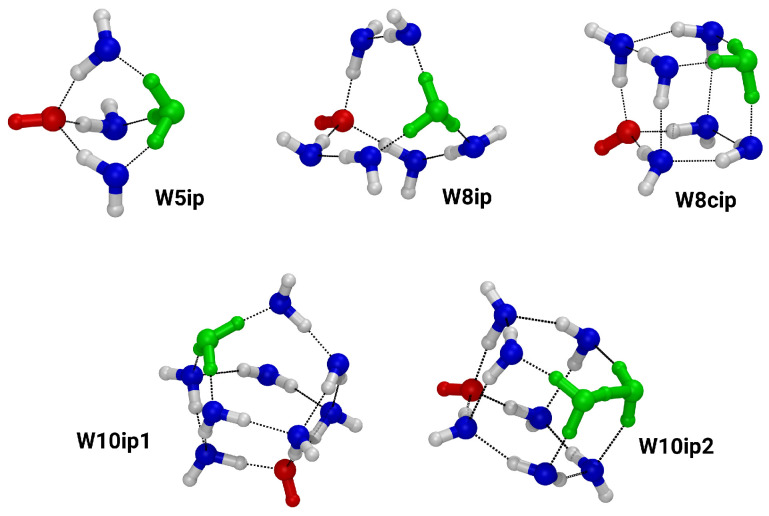
Ball-and-stick representation of the ion pair clusters. Oxygen: blue; hydrogen: white; red shows the hydroxide anions and green shows the hydronium cations.

Figure 2 shows the clusters that contain separated ion pairs, i.e., net neutral clusters with zwitterionic character. Almost all of them show the motif of the Eigen-ion (H_3_O^+^ donating three hydrogen bonds), except for the cluster W10ip2, which contains the Zundel ion motif (one H^+^ shared between two water molecules) [16]. The ion pair clusters range in size from a pentamer, W5ip, to decamer clusters, W10ip and W10ip2. Please note that the hydroxide anion always accepts three hydrogen bonds.

### 2.4. Quantum-Chemical Calculations

Geometry optimizations and vibrational frequency analyses were consistently performed using density functional theory with either the hybrid B3LYP [43,44] or PBE0 [45] density functional approximations and the triple-zeta def2-TZVP [46] basis set. To account for London dispersion interactions, the D3 dispersion correction with the Becke–Johnson damping function was applied [47]. Interaction energies of cluster structures are sensitive to the basis set superposition error (BSSE), which was corrected for by using a geometrical counterpoise correction (gCP) scheme [48]. This is essential, as proton transfer during cluster formation significantly alters the monomer geometries of a cluster, invalidating the assumptions of supramolecular approaches such as conventional counterpoise correction (CP) schemes [48]. As a third computational model, the composite density functional PBEh-3c was used, which includes both the gCP and D3 dispersion correction combined with a modified PBE hybrid functional and modified def2-SV(P) basis set, both optimized to maximize error cancellation [49].

Extensive studies on the importance of the underlying quantum chemical method to the quality of the QCE approach have been conducted in the past [34,50,51]. It was found that methods based on density functional theory are capable of producing results of similar quality to methods based on wave function theory. Recently, semi-empirical methods were shown to be a viable option if large-scale cluster sets are employed [52,53]. However, while some properties may be computed with reasonable accuracy for most investigated quantum chemical methods, others, such as the ionic product of water, were shown to be sensitive to the exact choice of method [20]. More details on the computational protocol can be found in our previous publication [20].

## 3. Results and Discussion

### 3.1. Variation in the QCE Parameters

In Table 1, optimized values of the QCE parameters, $a_{\mathrm{mf}}$ and $b_{\mathrm{xv}}$, are given for different density functional approximations, using standard QCE as well as mRRHO-QCE with switching function cutoff values of 50 and 100 cm^−1^.

There is little variation in $b_{\mathrm{xv}}$ across all methods, with values consistently lying in between 1.50 and 1.52. If all other parameters were kept the same, such a shift in $b_{\mathrm{xv}}$ would lead to a change in the computed density and temperature of phase transition of about −0.02 g cm^−1^ and 1.5 K, respectively. This very small dependence of $b_{\mathrm{xv}}$ on the method is expected, as this parameter corrects the translational volume for the eigenvolume of the clusters and thus depends neither on the quantum-chemical description of the clusters nor on the precise details of the vibrational partition function.

The mean-field parameter, $a_{\mathrm{mf}}$, in contrast, varies between 0.17 and 0.22 J m^3^ mol^−2^. Again, if all other parameters were kept the same, such a shift in $a_{\mathrm{mf}}$ would lead to an increase in the computed temperature of phase transition of about 24.5 K, whereas the computed density remains almost the same, increasing only by 0.01 g cm^−1^. Its value depends significantly on the functional, increasing in the order PBEh-3c < PBE0/D3/gCP < B3LYP/D3/gCP. Since $a_{\mathrm{mf}}$ accounts for interactions between clusters, the parameter has in the past been observed to compensate for underbinding of the electronic structure method, in which case $a_{\mathrm{mf}}$ values become larger. Increasing the cutoffs for the mRRHO model also leads to larger values of $a_{\mathrm{mf}}$, indicating an indirect impact of the treatment of these internal rotations on the mean-field energy. The vibrational motions at low frequencies are usually internal rotations or translations, and hence can be related to the process of cluster association. It is therefore reasonable that the mean-field parameter $a_{\mathrm{mf}}$ is sensitive to the particular form of the vibrational partition function for these modes, while $b_{\mathrm{xv}}$ is not.

### 3.2. Ionic Product Dependence on mRRHO

Given the populations of the ion pair clusters, it is easily possible to compute the ionic product [20],

(10) Kw=cH+·cOH−=∑℘∈ionpairclustersN℘2V2,

with $c_{H^{+}}$ and $c_{{\mathrm{OH}}^{-}}$ referring to the proton and hydroxide ion concentrations, respectively, and exploiting the fact that there is exactly one ion pair in every cluster investigated in this study.

**Figure 3:**
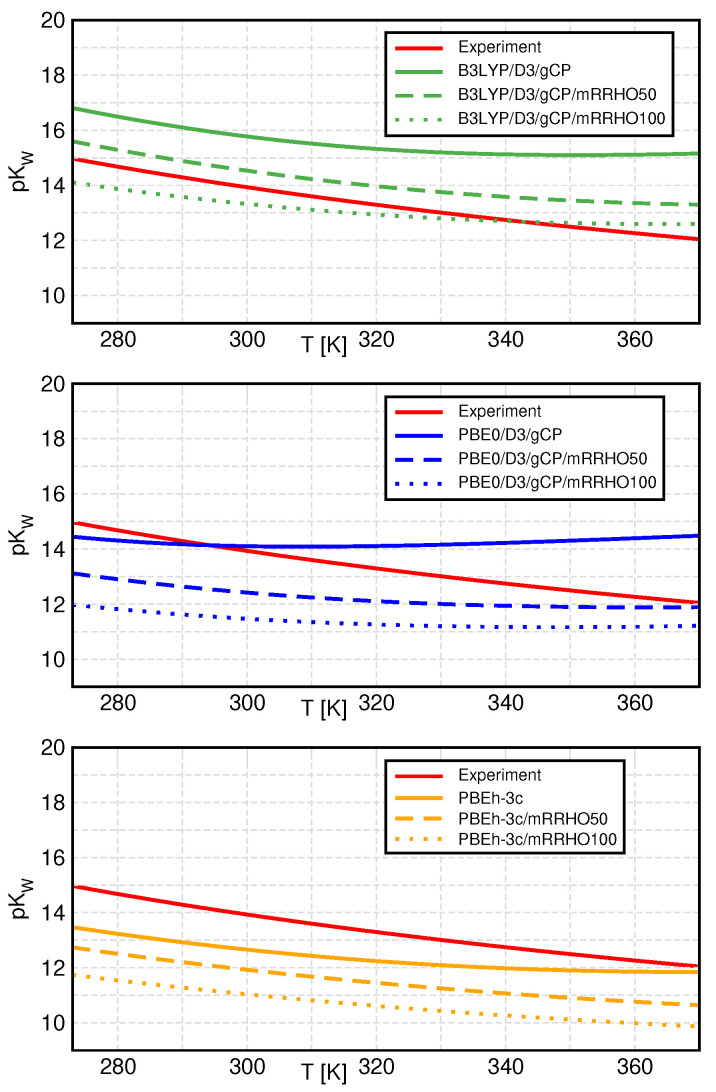
Temperature dependence of the negative logarithm of the ionic product, $pK_{w}$, for all selected methods as well as experimental [54] values. The standard method is represented by solid, mRRHO50 by dashed, and mRRHO100 by dotted lines.

In Figure 3, we compare the temperature dependence of $pK_{w}$ between standard QCE and mRRHO-QCE for all three investigated methods (top: B3LYP, middle: PBE0, bottom: PBEh-3c, all with D3 and gCP). In general, the application of mRRHO leads to decreasing values of $pK_{w}$, which translates to an increasing ionic product, and hence, larger ion concentrations in the liquid phase. Consequently, we see an improvement in the results for B3LYP, which predicted slightly too large values for $pK_{w}$ in the standard QCE model. PBE0, which produced the best data in the standard QCE approach, is now underestimating $pK_{w}$. The same is observed for PBEh-3c, which already resulted in the smallest values within the standard RRHO model.

The temperature dependence of $pK_{w}$ is also altered by the introduction of the mRRHO approach. The observed reduction in $pK_{w}$ from standard QCE (solid curves) to mRRHO50 (dashed curves) is largest at high temperatures and slightly smaller at low temperatures, corresponding to a smaller slope of the curves. Changing the mRRHO cutoff value to 100 cm^−1^ leads to similar, but less pronounced changes, except for PBE0, where the slope of $pK_{w}$ is sightly increased again.

In Table 2, we list the number of low-frequency modes per cluster that are subject to the modified partition function for the B3LYP/D3/gCP method. The full list of vibrational modes for all methods is given in Table S1 in the Supporting Information. Only 27 vibrations are affected by the mRRHO50 approach, compared to as many as 86 vibrations in the mRRHO100 model. Still, the impact on the ionic product is more pronounced in the former case, indicating that $K_{W}$ is affected most by low-frequency modes.

**Figure 4:**
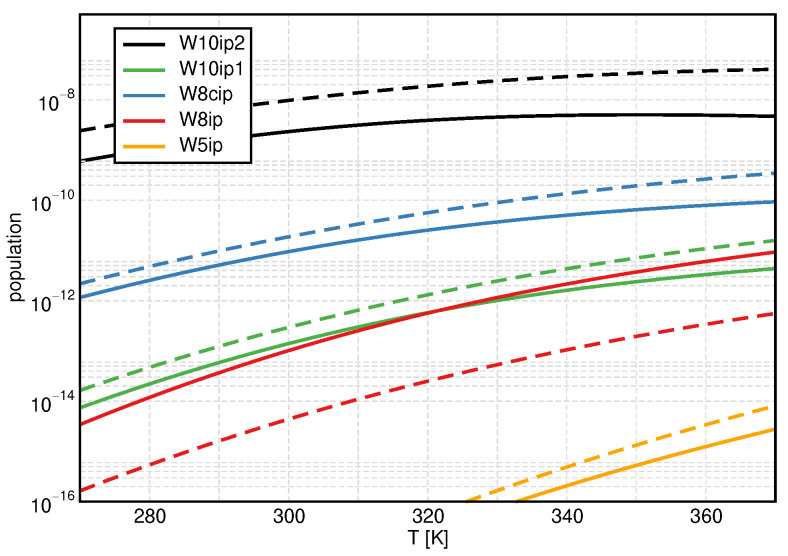
Temperature dependence of the monomer-normalized populations for the ion pair clusters, showing B3LYP/D3/gCP data as a representative example. Solid lines: conventional QCE, dashed lines: mRRHO50.

**Figure 5:**
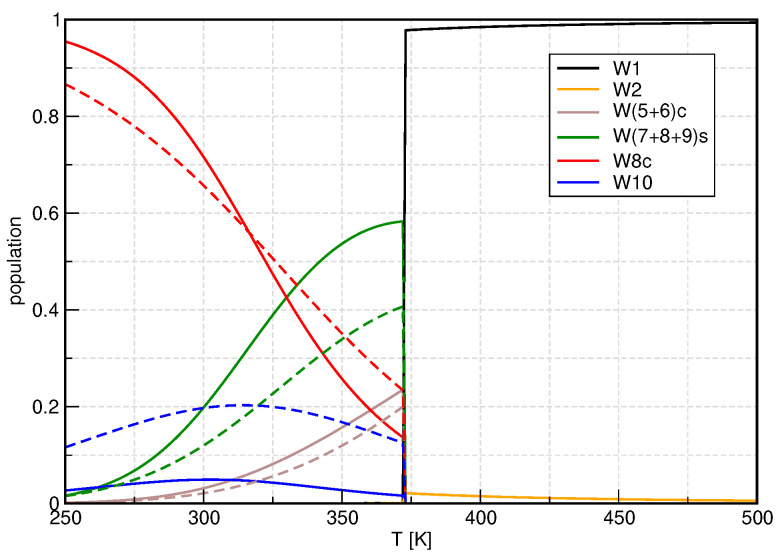
Temperature dependence of the monomer-normalized populations for the regular clusters, showing B3LYP/D3/gCP data as a representative example. Solid lines: conventional QCE, dashed lines: mRRHO50. Note the semilogarithmic scale.

To substantiate our observations for the ionic product, we consider the populations of ion pair clusters in Figure 4 and regular clusters in Figure 5, using once again B3LYP/D3/gCP as a representative example (see Figures S1 and S2 for results obtained with mRRHO100). All ion pair clusters but W8ip show increased populations if treated with the mRRHO50 approach. The population of W8ip is significantly reduced. It is interesting to note that W8ip shows three modes below 50 cm^−1^ while the others exhibit none or only two (see Table 2). Keeping in mind that the y-axis has a logarithmic scale, it is clear that W10ip2 is the dominating ion pair cluster, causing the overall increase in $pK_{w}$.

While the ion pair clusters are essential for the calculation of the ionic product, they hardly contribute to macroscopic thermodynamic quantities of liquid water, which are determined by the regular clusters, instead. In Figure 5, we show the populations of those clusters, grouped by the structural motifs (monomer, dimer, cyclic clusters, spiro clusters, cubelike, sandwich-shaped). Since the monomer and dimer do not exhibit any vibrational modes lower than 100 cm^−1^, the populations of these clusters are not influenced by the modified vibrational partition function. The populations of clusters that are dominated by ring structures, be it cyclic clusters or condensed rings in spiroclusters, are reduced if the mRRHO50 model is employed.

Considering the number of low-frequency modes (Table 2), W6c, W7, and W9 contain (many) modes below 50 cm^−1^ and their individual population is reduced. However, W5c and W8b also show these modes and obtain slightly higher or unaltered populations. For the sandwich-type decamer, the population is significantly increased if the hindered rotor model is used to treat internal rotations. Please note that this cluster shows no modes below 50 cm^−1^, see Table 2. While it is still not the dominant cluster in the liquid phase, its population is enhanced by a factor of around four so that within the mRRHO50 model up to 20% of all water molecules are arranged in this shape. Interestingly, the population of the cubic W8c cluster is decreased at low temperatures and increased at higher temperatures.

### 3.3. Clausius–Clapeyron Analysis

Motivated by the capability of the QCE approach to treat phase transitions and the option to apply different pressures, we performed a Clausius–Clapeyron analysis following the original work of Weinhold [30]. QCE calculations with the parameters listed in Table 1 have been conducted at different pressures ranging from 0.095898 bar to 4.7572 bar and the observed temperatures of phase transition have been noted, see Table S2 of the Supporting Information.

Overall, we observe that the temperatures of phase transition deviate only slightly between standard QCE and either of the mRRHO approaches. The maximum deviation between the approaches is 3 K in the investigated pressure range. A similar observation is made for the different density functional approximations, with phase transition temperature variations of up to only 5 K. Of course, since the parameters have been optimized at the standard pressure of 1.01325 bar, deviations at very small and very large pressures are larger than at the reference pressure.

**Figure 6:**
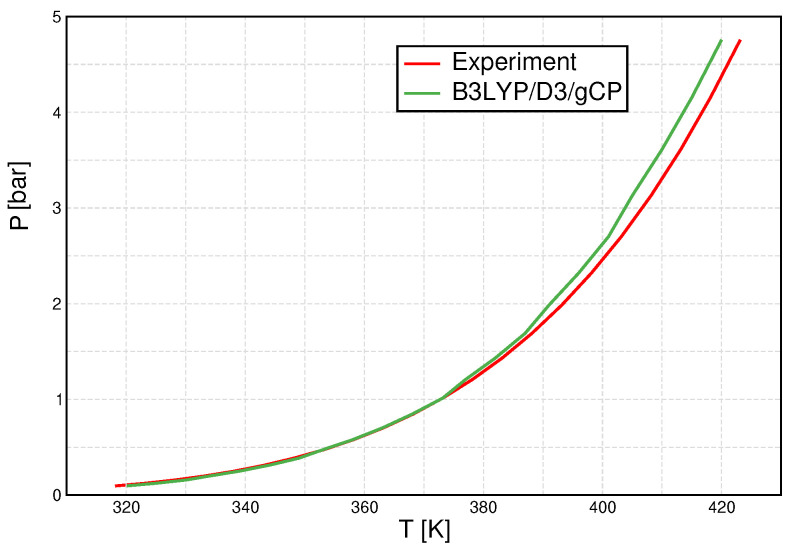
Pressure–temperature graph for B3LYP/D3/gCP (green line) and compared to experimental [55] data (red line).

Once again, we show B3LYP/D3/gCP data as an example (Figure 6). The agreement between calculated and experimental data is astonishing, especially recalling the finite cluster set and the fact that only two parameters are optimized to reproduce one of these data points. There is some deviation in the high-temperature/high-pressure regime, which can, however, still be considered to be reasonable agreement.

**Figure 7:**
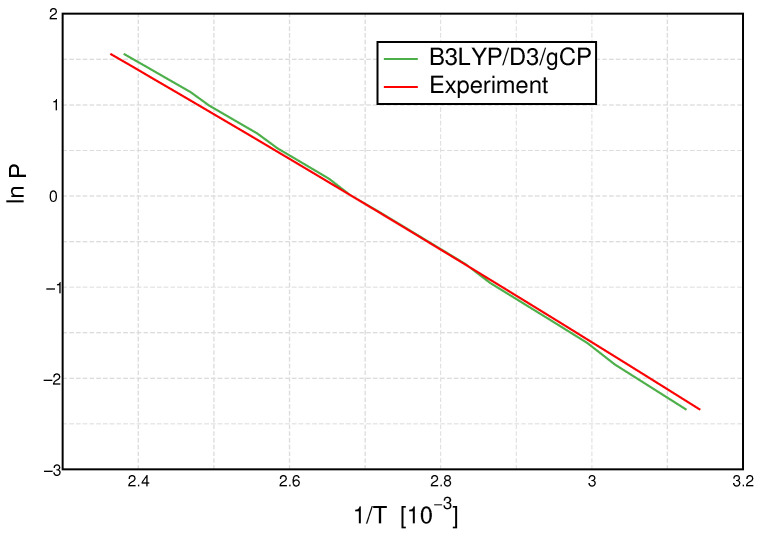
Clausius–Clapeyron plot: Logarithmic plot of pressure P versus inverse temperature T for B3LYP/D3/gCP (green line) and experiment [55] (red line).

The integrated form of the Clausius–Clapeyron relation states that the natural logarithm of the pressure is proportional to the inverse temperature of the phase transition, which is why we show lnP vs. $T^{-1}$ in Figure 7. In this plot, rather small differences in the linear graph in Figure 6 at small temperatures and pressures are amplified, now occurring in the bottom right of the plot.

Naturally, and due to the parameter-optimization procedure, a perfect agreement at ambient pressure (lnP∼0) is observed, while the slight deviations from the experiment have a comparable magnitude below and above that point. With the Clausius–Clapeyron relation, an estimate of the enthalpy of vaporization $\Delta H_{\mathrm{vap}}$ can be deduced from the slope of these curves. The resulting calculated enthalpies are listed and compared to the experimental value in Table 3. As already visible from Figure 7, the agreement of the calculated values with the experimental reference is excellent. While all calculations overestimate the enthalpy of vaporization, a deviation of only 2 kJ mol^−1^ is observed for PBE0/D3/gCP. All mRRHO calculations lead to an overestimation of this quantity, whereas the B3LYP data are least sensitive to the mRRHO approach and yield the best overall data, which is in agreement with the observations made for the ionic product. With the exception of PBEh-3c, all methods reproduce the experimental enthalpy of vaporization to within 4 kJ mol^−1^, a threshold often referred to as a targeted chemical accuracy.

### 3.4. Entropy

**Figure 8:**
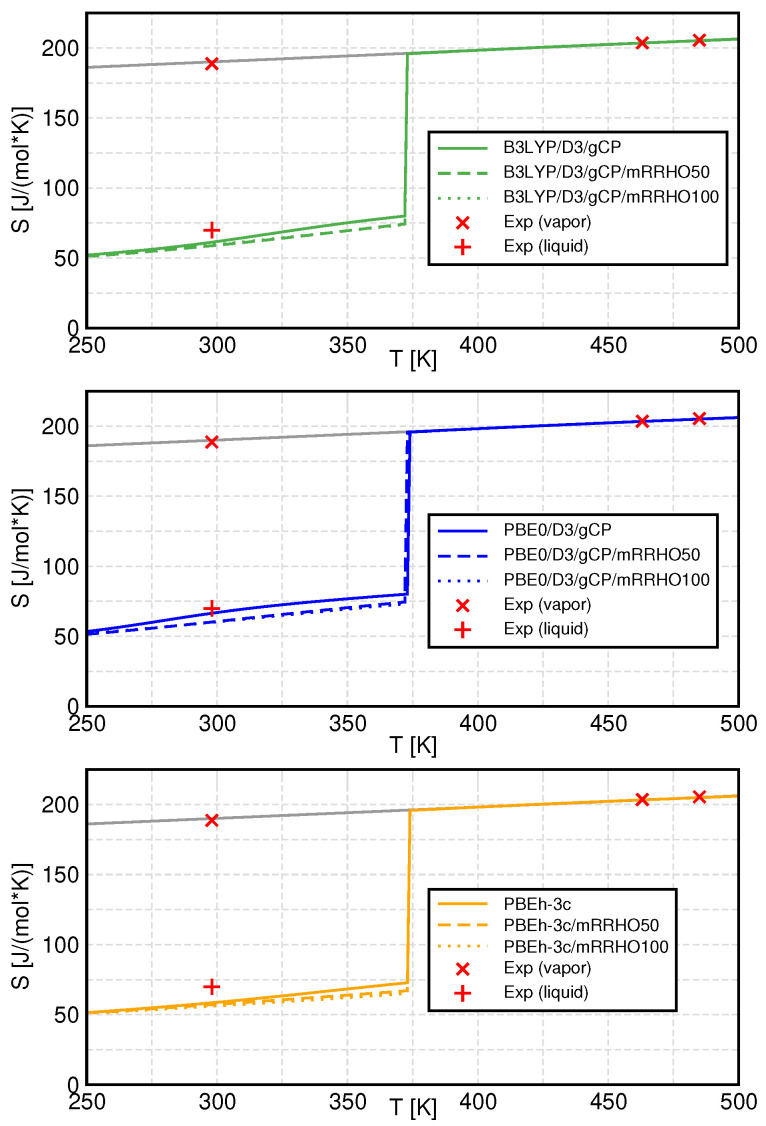
Temperature dependence of the entropy for all methods (colored lines) as well as experimental values (red marks). Standard QCE results are presented as solid, mRRHO50 results as dashed, and mRRHO100 results as dotted lines. The grey line is a linear extrapolation of the gas-phase curve. Experimental values are from Refs. [57,58,59].

Finally, the entropy of liquid water and water vapor in the liquid range and beyond the phase transition point are presented for the different methods and mRRHO treatments. Calculated and experimental values for the entropy are shown in Figure 8 and phase change data are listed in Table 3.

It can be seen that the gas-phase entropy is hardly affected by any variation of either the quantum-chemical methods or the models for the vibrational partition function. This is plausible, since the gas phase is mainly composed of monomer units and a small number of dimers, both of which are unaffected by the mRRHO approach since they do not exhibit low-frequency vibrations. Furthermore, at such large phase volumes, the translational contributions become dominant which leads to a negligible influence of the electronic structure method.

In the liquid phase and at lower temperatures, the contributions of the individual clusters and the treatment of internal rotations become more important, leading to differences between the density functionals and between the mRRHO treatments. All three functionals underestimate the entropy at ambient temperature (298 K), and therefore, overestimate the entropy of vaporization at that temperature. This is even more pronounced if mRRHO is applied, which leads to even smaller liquid phase entropies in all cases. The effect of the cutoff value has a minor influence here, with mRRHO100 values being only slightly smaller than for mRRHO50.

## 4. Conclusions

In this contribution, we combined Frank Weinhold’s QCE theory with a treatment of low-frequency vibrational modes as hindered rotations rather than harmonic vibrations (mRRHO). The approach has been applied to the characterization of water, for which we computed a diverse set of properties, ranging from the microscopic effect of self-dissociation to macroscopic thermodynamic data over a range of pressures and temperatures.

We observed a significant influence of the mRRHO model on the cluster populations and derived properties such as the ionic product. The effect on thermodynamic quantities was present, but less pronounced. Since the relationship between the individual cluster partition functions and the final thermodynamic quantities of the associated phase is very complex, no clear predictions can be made in terms of which properties are affected most, how the properties are affected, or what the best cutoff value is. The influence of the mRRHO approach does indicate, however, that improving the computational chemistry tool set beyond the RRHO approximation is key to the successful description of thermodynamic data and should continue to be addressed in future studies.

The performance of QCE calculations at different pressures, and thereby, the prediction of phase-change data using the Clausius–Clapeyron relation is compelling. With the small cluster set presented in this study and by optimizing only two empirical parameters, we were able to reproduce the enthalpy of vaporization with chemical accuracy.

The QCE approach has been applied to various systems in the past (see Ref. [60]), and its areas of application are continuously being extended. Recent examples include protic ionic liquids [61], aqueous solutions of weak acids [31,32], and various organic solvents [38]. The mRRHO model has led to significant improvements in the treatment of weakly interacting aprotic compounds such as methane and chloroform. While its applicability is currently restricted to fluid phases, investigations of critical phase systems or of solids dissolved in liquids are conceivable and are possible subjects of future studies.

Once more, we conclude that the QCE approach—despite its simplicity and limited cluster set—is capable of providing a surprisingly accurate description of a complicated liquid such as water. All investigated quantities could be reproduced with astonishing agreement with the experimental reference data, rendering the QCE approach a powerful tool for future investigations of associated liquids in various contexts.

## Figures and Tables

**Table 1:** Optimized QCE parameters $a_{\mathrm{mf}}$ in $J\;m^{3}\;{\mathrm{mol}}^{-2}$ and $b_{\mathrm{xv}}$ (dimensionless) for different methods.

Method	Standard QCE		mRRHO50		mRRHO100	
	$a_{\mathrm{mf}}$	$b_{\mathrm{xv}}$	$a_{\mathrm{mf}}$	$b_{\mathrm{xv}}$	$a_{\mathrm{mf}}$	$b_{\mathrm{xv}}$
B3LYP/D3/gCP	0.201	1.50	0.213	1.50	0.216	1.50
PBE0/D3/gCP	0.192	1.52	0.205	1.50	0.207	1.50
PBEh-3c	0.173	1.50	0.185	1.52	0.187	1.52

**Table 2:** Number of vibrational modes below 50 cm^−1^ and between 50 cm^−1^ and 100 cm^−1^, respectively, for selected clusters from Figure 1 and Figure 2 for the B3LYP/D3/gCP method.

Cluster	# Modes $\tilde{\nu }<50\;{\mathrm{cm}}^{-1}$	# Modes $50\;{\mathrm{cm}}^{-1}<\tilde{\nu }<100\;{\mathrm{cm}}^{-1}$
W5p	1	2
W5c	2	2
W6c	2	4
W7	3	4
W8p	4	5
W8b	4	4
W8c	−	5
W9	6	5
W10	−	7
W5ip	−	1
W8ip	3	5
W8cip	−	3
W10ip1	2	6
W10ip2	−	6

**Table 3:** First line: enthalpy of vaporization $\Delta H_{\mathrm{vap}}$ in kJ mol^−1^ calculated through a Clausius–Clapeyron analysis. The experimental value is based on the data shown in the SI from Ref. [56]. Next lines: entropy of vaporization in J mol^−1^ K^−1^ at 298 K and at the temperatures of phase transition (373 K). See Section 3.4 for details. Experimental values are from Refs. [57,58,59].

	B3LYP/D3/gCP			PBE0/D3/gCP			PBEh-3c			Exp
mRRHO	−	50	100	−	50	100	−	50	100	
$\Delta H_{\mathrm{vap}}$	43.74	45.89	45.93	43.57	45.61	46.26	46.31	48.20	49.03	41.58
$\Delta S_{\mathrm{vap}}$(298 K)	128.69	131.21	130.68	123.45	129.70	129.92	131.47	132.82	133.90	118.76
$\Delta S_{\mathrm{vap}}$(trs)	115.78	121.46	121.73	115.64	121.21	122.37	122.97	128.62	130.51	109.54

## Data Availability

All calculated data are available upon request from the authors.

## Supplementary Materials

The following are available online. Figure S1: mRRHO100-QCE populations of ion pair clusters; Figure S2: mRRHO100-QCE populations of regular clusters Title; Figure S3: Comparison of $pK_{w}$ with different parameter optimization schemes; Table S1: Low lying vibrational modes for selected clusters; Table S2: QCE phase transition temperatures at different pressures.

## References

[1] Cukierman S. (2006). Et tu, Grotthuss! and other unfinished stories. Biochim. Biophys. Acta Bioenerg..

[2] Grotthuss C.J.T.d. (2006). Memoir on the decomposition of water and of the bodies that it holds in solution by means of galvanic electricity. Biochim. Biophys. Acta Bioenerg..

[3] Pauliukaite R., Juodkazytė J., Ramanauskas R. (2017). Theodor von Grotthuss’ Contribution to Electrochemistry. Electrochim. Acta.

[4] Clausius R. (1857). Ueber die Elektricitätsleitung in Elektrolyten. Ann. Phys..

[5] Danneel H. (1905). Notiz über Ionengeschwindigkeiten. Ber. Bunsen-Ges. Phys. Chem..

[6] Ädelroth P. (2006). Special issue on proton transfer in biological systems. Biochim. Biophys. Acta Bioenerg..

[7] Zhang J., Zhang M., Tang K., Verpoort F., Sun T. (2014). Polymer-Based Stimuli-Responsive Recyclable Catalytic Systems for Organic Synthesis. Small.

[8] Tang H., Zhao W., Yu J., Li Y., Zhao C. (2019). Recent Development of pH-Responsive Polymers for Cancer Nanomedicine. Molecules.

[9] Liu J., Qu S., Suo Z., Yang W. (2020). Functional hydrogel coatings. Natl. Sci. Rev..

[10] Dai S., Ravi P., Tam K.C. (2008). pH-Responsive polymers: Synthesis, properties and applications. Soft Matter.

[11] Kocak G., Tuncer C., Bütün V. (2016). pH-Responsive polymers. Polym. Chem..

[12] Tuckerman M., Laasonen K., Sprik M., Parrinello M. (1995). Ab initio molecular dynamics simulation of the solvation and transport of hydronium and hydroxyl ions in water. J. Chem. Phys..

[13] Marx D., Tuckerman M.E., Hutter J., Parrinello M. (1999). The nature of the hydrated excess proton in water. Nature.

[14] Geissler P.L., Voorhis T.V., Dellago C. (2000). Potential energy landscape for proton transfer in (H_2_O)_3_H^+^: Comparison of density functional theory and wavefunction-based methods. Chem. Phys. Lett..

[15] Geissler P.L., Dellago C., Chandler D., Hutter J., Parrinello M. (2001). Autoionization in Liquid Water. Science.

[16] Kirchner B. (2007). Eigen or Zundel Ion: News from Calculated and Experimental Photoelectron Spectroscopy. ChemPhysChem.

[17] Hassanali A., Prakash M.K., Eshet H., Parrinello M. (2011). On the recombination of hydronium and hydroxide ions in water. Proc. Natl. Acad. Sci. USA.

[18] Chandler D., Dellago C., Geissler P. (2012). Wired-up water. Nat. Chem..

[19] Sakti A.W., Nishimura Y., Chou C.P., Nakai H. (2018). Density-Functional Tight-Binding Molecular Dynamics Simulations of Excess Proton Diffusion in Ice Ih, Ice Ic, Ice III, and Melted Ice VI Phases. J. Phys. Chem. A.

[20] Perlt E., von Domaros M., Kirchner B., Ludwig R., Weinhold F. (2017). Predicting the ionic product of water. Sci. Rep..

[21] Sprik M. (2000). Computation of the pK of liquid water using coordination constraints. Chem. Phys..

[22] Himmel D., Goll S.K., Leito I., Krossing I. (2012). Bulk Gas-Phase Acidity. Chem. Eur. J..

[23] Sato H., Hirata F. (1998). Theoretical Study for Autoionization of Liquid Water: Temperature Dependence of the Ionic Product (pK w). J. Phys. Chem. A.

[24] Sato H., Hirata F. (1999). Ab Initio Study on Molecular and Thermodynamic Properties of Water: A Theoretical Prediction of pK w over a Wide Range of Temperature and Density. J. Phys. Chem. B.

[25] Klamt A., Eckert F., Diedenhofen M., Beck M.E. (2003). First Principles Calculations of Aqueous pK a Values for Organic and Inorganic Acids Using COSMO–RS Reveal an Inconsistency in the Slope of the pK a Scale. J. Phys. Chem. A.

[26] Yoshida N., Ishizuka R., Sato H., Hirata F. (2006). Ab Initio Theoretical Study of Temperature and Density Dependence of Molecular and Thermodynamic Properties of Water in the Entire Fluid Region: Autoionization Processes. J. Phys. Chem. B.

[27] Pereira R.W., Ramabhadran R.O. (2020). pK-Yay: A Black-Box Method Using Density Functional Theory and Implicit Solvation Models to Compute Aqueous pK a Values of Weak and Strong Acids. J. Phys. Chem. A.

[28] Pongratz T., Kibies P., Eberlein L., Tielker N., Hölzl C., Imoto S., Erlach M.B., Kurrmann S., Schummel P.H., Hofmann M. (2020). Pressure-dependent electronic structure calculations using integral equation-based solvation models. Biophys. Chem..

[29] Weinhold F. (1998). Quantum cluster equilibrium theory of liquids: General theory and computer implementation. J. Chem. Phys..

[30] Weinhold F. (1998). Quantum cluster equilibrium theory of liquids: Illustrative application to water. J. Chem. Phys..

[31] Blasius J., Ingenmey J., Perlt E., von Domaros M., Hollóczki O., Kirchner B. (2019). Predicting mole-fraction-dependent dissociation for weak acids. Angew. Chem. Int. Ed..

[32] Blasius J., Ingenmey J., Perlt E., von Domaros M., Hollóczki O., Kirchner B. (2019). Dissoziation schwacher Säuren über den gesamten Molenbruchbereich. Angew. Chem..

[33] Grimme S. (2012). Supramolecular Binding Thermodynamics by Dispersion-Corrected Density Functional Theory. Chem. Eur. J..

[34] Kirchner B. (2005). Cooperative versus dispersion effects: What is more important in an associated liquid such as water?. J. Chem. Phys..

[35] Kirchner B., Spickermann C., Lehmann S.B., Perlt E., Langner J., von Domaros M., Reuther P., Uhlig F., Kohagen M., Brüssel M. (2011). What can clusters tell us about the bulk?: Peacemaker: Extended quantum cluster equilibrium calculations. Comput. Phys. Commun..

[36] Brüssel M., Perlt E., Lehmann S.B.C., von Domaros M., Kirchner B. (2011). Binary systems from quantum cluster equilibrium theory. J. Chem. Phys..

[37] von Domaros M., Perlt E., Ingenmey J., Marchelli G., Kirchner B. (2018). Peacemaker2: Making clusters talk about binary mixtures and neat liquids. SoftwareX.

[38] Zaby P., Ingenmey J., Kirchner B., Grimme S., Ehlert S. (2021). Calculation of improved enthalpy and entropy of vaporization by a modified partition function in quantum cluster equilibrium theory. J. Chem. Phys..

[39] McQuarrie D., Simon J., Cox H., Simon D., Choi J. (1997). Physical Chemistry: A Molecular Approach.

[40] Nelder J.A., Mead R. (1965). A simplex method for function minimization. Comput. J..

[41] Marchelli G., Ingenmey J., Hollóczki O., Chaumont A., Kirchner B. (2021). Hydrogen Bonding and Vaporization Thermodynamics in Hexafluoroisopropanol-Acetone and-Methanol Mixtures. A Joined Cluster Analysis and Molecular Dynamic Study. ChemPhysChem.

[42] Chai J.D., Head-Gordon M. (2008). Long-range corrected hybrid density functionals with damped atom–atom dispersion corrections. Phys. Chem. Chem. Phys..

[43] Becke A.D. (1988). Density-functional exchange-energy approximation with correct asymptotic behavior. Phys. Rev. A.

[44] Lee C., Yang W., Parr R.G. (1988). Development of the Colle-Salvetti correlation-energy formula into a functional of the electron density. Phys. Rev. B.

[45] Adamo C., Barone V. (1999). Toward reliable density functional methods without adjustable parameters: The PBE0 model. J. Chem. Phys..

[46] Weigend F., Ahlrichs R. (2005). Balanced basis sets of split valence, triple zeta valence and quadruple zeta valence quality for H to Rn: Design and assessment of accuracy. Phys. Chem. Chem. Phys..

[47] Grimme S., Ehrlich S., Goerigk L. (2011). Effect of the damping function in dispersion corrected density functional theory. J. Comput. Chem..

[48] Kruse H., Grimme S. (2012). A geometrical correction for the inter- and intra-molecular basis set superposition error in Hartree-Fock and density functional theory calculations for large systems. J. Chem. Phys..

[49] Grimme S., Brandenburg J.G., Bannwarth C., Hansen A. (2015). Consistent structures and interactions by density functional theory with small atomic orbital basis sets. J. Chem. Phys..

[50] Perlt E., Friedrich J., von Domaros M., Kirchner B. (2011). Importance of Structural Motifs in Liquid Hydrogen Fluoride. ChemPhysChem.

[51] Spickermann C., Perlt E., von Domaros M., Roatsch M., Friedrich J., Kirchner B. (2011). Coupled Cluster in Condensed Phase. Part II: Liquid Hydrogen Fluoride from Quantum Cluster Equilibrium Theory. J. Chem. Theory Comput..

[52] Marchelli G., Ingenmey J., Kirchner B. (2020). Activity coefficients of binary methanol alcohol mixtures from cluster weighting. ChemistryOpen.

[53] Ingenmey J., Blasius J., Marchelli G., Riegel A., Kirchner B. (2019). A Cluster Approach for Activity Coefficients: General Theory and Implementation. J. Chem. Eng. Data.

[54] Marshall W.L., Franck E.U. (1981). Ion product of water substance, 0-1000 C, 1-10,000 bars. New International Formulation and its background. J. Phys. Chem. Ref. Data.

[55] Wagner W., Pruß A. (2002). The IAPWS Formulation 1995 for the Thermodynamic Properties of Ordinary Water Substance for General and Scientific Use. J. Phys. Chem. Ref. Data.

[56] Haynes W.M. (2014). CRC Handbook of Chemistry and Physics.

[57] Chase M.W.J. (1998). NIST-JANAF Themochemical Tables, Fourth Edition. J. Phys. Chem. Ref. Data Monogr..

[58] Giauque W.F., Archibald R.C. (1937). The Entropy of Water from the Third Law of Thermodynamics. The Dissociation Pressure and Calorimetric Heat of the Reaction Mg(OH)_2_ = MgO + H_2_O. The Heat Capacities of Mg(OH)_2_ and MgO from 20 to 300°K. J. Am. Chem. Soc..

[59] McBride B., Gordon S. (1961). Thermodynamic functions of several triatomic molecules in the ideal gas state. J. Chem. Phys..

[60] Kirchner B. (2007). Theory of complicated liquids: Investigation of liquids, solvents and solvent effects with modern theoretical methods. Phys. Rep..

[61] Ingenmey J., von Domaros M., Perlt E., Verevkin S.P., Kirchner B. (2018). Thermodynamics and proton activities of protic ionic liquids with quantum cluster equilibrium theory. J. Chem. Phys..

