# Supplementary material for: The Ionic Product of Water in the Eye of the Quantum Cluster Equilibrium
Source: Molecules. 2022 Feb 14;27(4):1286. doi: 10.3390/molecules27041286 (PMC8877775; doi:10.3390/molecules27041286)
Supplement: Supplementary file 1 [file molecules-27-01286-s001.zip › molecules-1559781-supplementary.pdf]

Supplementary Materials: The Ionic Product of Water in the Eye of the Quantum Cluster Equilibrium

Barbara Kirchner, Johannes Ingenmey, Michael von Domaros and Eva Perlt

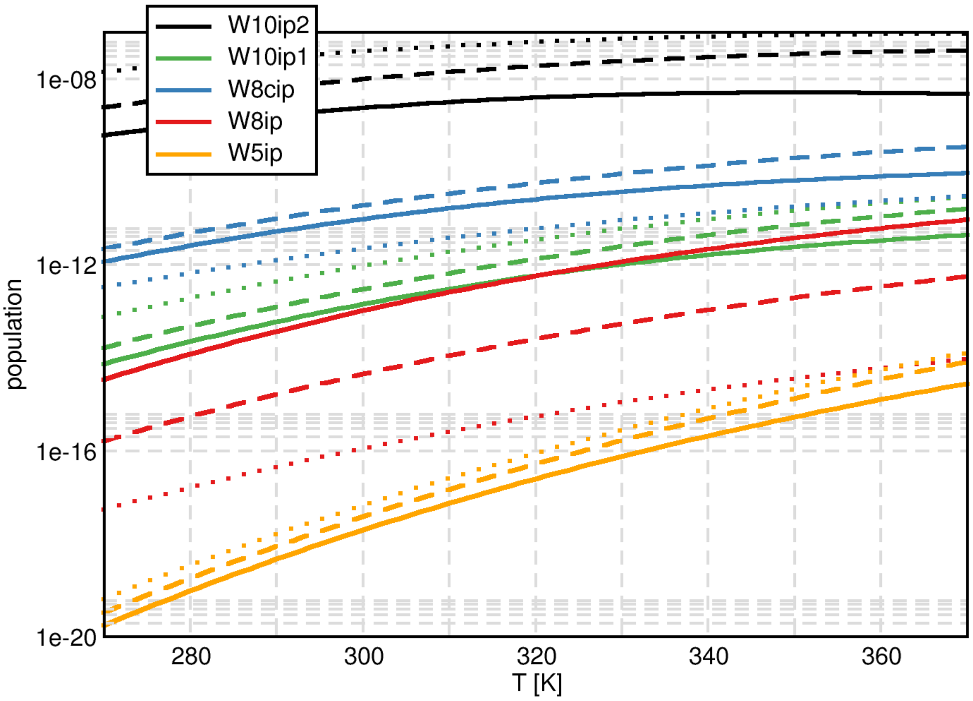

Figure S1. Temperature dependence of the monomer-normalized populations of the ion pair clusters. The y-axis shows, on logarithmic scale, the data for the B3LYP/D3/gCP method, using standard QCE (solid line), mRRHO50 (dashed line), and mRRHO100 (dotted line).

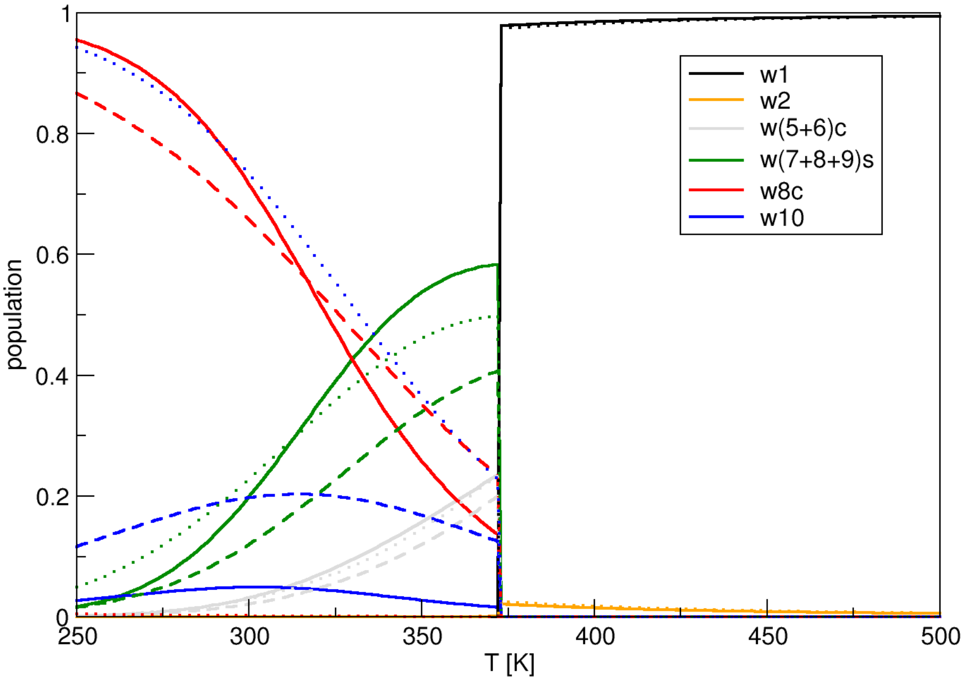

Figure S2. Temperature dependence of the monomer-normalized populations of the regular clusters, showing B3LYP/D3/gCP data as a representative example. Solid lines: conventional QCE, dashed lines: mRRHO50, and dotted lines: mRRHO100.

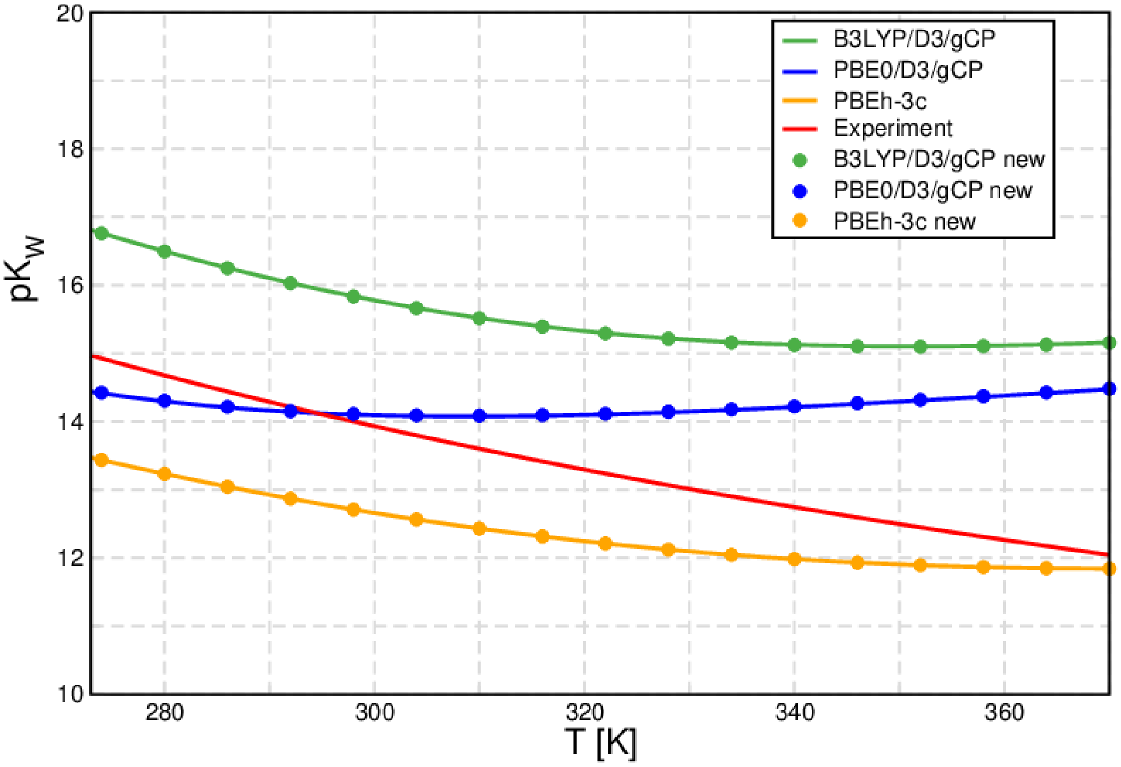

Figure S3. Temperature dependence of the negative logarithm of the ionic product, pK_W. Comparing results from our previous study [1] (lines) and to those obtained here, using the novel parameter optimization algorithm (circles) as well as experimental [2] values. The perfect agreement of both calculations underline the reliability and reproducibility of QCE data as well as numerical stability and robustness of the Peacemaker code. [3–5]

Table S1: Low lying vibrational modes for selected clusters shown in Figures 1 and 2 in the manuscript for the B3LYP/D3/gCP method.

cluster	$\tilde{\nu} < 50$ cm^{-1}		$50 < \tilde{\nu} < 100$ cm^{-1}	
**W5p**	11.54		64.19	96.24
**W5c**	22.10	44.74	69.48	73.14
**W6c**	30.11	31.19	50.12<br>55.06	50.20<br>91.88
**W7**	16.92<br>36.45	19.75	58.33<br>66.96	59.11<br>94.02
**W8p**	26.41<br>37.61	34.03<br>42.44	50.80<br>62.19<br>97.74	58.20<br>88.38
**W8b**	27.34<br>36.73	30.47<br>45.72	50.44<br>84.51	82.76<br>86.98
**W8c**	–		78.33<br>82.33<br>84.95	80.62<br>84.83
**W9**	13.87<br>27.83<br>43.89	21.09<br>42.54<br>47.49	57.64<br>73.91<br>88.84	70.68<br>78.99
**W10**	–		60.65<br>65.12<br>69.34<br>76.26	61.08<br>65.79<br>72.55
**W5ip**	–<br>38.85	 39.38	90.71<br>55.85	 55.90
**W8ip**	39.66		85.43<br>89.36	85.51
**W8cip**	–		86.32<br>97.50	86.75
**W10ip1**	22.31	44.28	58.79<br>69.58<br>84.72	67.54<br>80.24<br>98.85
**W10ip2**	–		65.69<br>75.12<br>84.17	69.90<br>80.80<br>96.46

Table S2: Temperatures of phase transition, $T^{\star}$, in K for different pressures, P, in bar, different methods and different cutoff values for the mRRHO approach (a dash indicates the harmonic approximation) determined according to Ref. [6]. Experimental values from Ref. [7].

	B3LYP/D3/gCP			PBE0/D3/gCP			PBEh-3c			Exp
mRRHO	–	50	100	–	50	100	–	50	100	
0.095898	320	322	323	320	322	322	322	324	325	318.15
0.12344	325	327	327	325	326	327	327	329	329	323.15
0.15752	330	331	332	330	330	331	332	333	334	328.15
0.19932	334	336	337	334	336	336	336	338	338	333.15
0.25022	339	340	341	339	340	341	341	342	343	338.15
0.31176	344	345	346	344	345	345	345	347	347	343.15
0.38563	349	350	351	349	350	350	350	351	351	348.15
0.47373	353	354	355	354	354	354	355	356	356	353.15
0.57815	358	359	360	359	359	359	359	360	360	358.15
0.70117	363	363	364	364	363	363	364	365	364	363.15
0.84529	368	368	369	368	368	368	368	369	369	368.15
1.01325	373.15	373.15	373.15	373.15	373.15	373.15	373.15	373.15	373.15	373.15
1.2079	377	377	378	378	377	377	377	377	377	378.15
1.4324	382	381	382	384	381	381	381	382	381	383.15
1.6902	387	386	387	387	386	386	386	386	385	388.15
1.9848	391	390	391	392	390	390	390	390	389	393.15
2.3201	396	395	396	397	395	394	395	394	394	398.15
2.7002	401	399	400	401	399	399	399	398	398	403.15
3.1293	405	404	405	406	404	403	403	402	402	408.15
3.6119	410	408	409	410	408	407	408	406	406	413.15
4.1529	415	412	414	415	412	412	412	411	410	418.13
4.7572	420	417	418	420	417	416	416	415	413	423.15

1. Perlt, E.; von Domaros, M.; Kirchner, B.; Ludwig, R.; Weinhold, F. Predicting the ionic product of water. *Sci. Rep.* **2017**, *7*, 1–10.
2. Marshall, W.L.; Franck, E.U. Ion product of water substance, 0-1000 C, 1-10,000 bars. New International Formulation and its background. *J. Phys. Chem. Ref. Data* **1981**, *10*, 295–304.
3. Kirchner, B. Cooperative versus dispersion effects: What is more important in an associated liquid such as water? *J. Chem. Phys.* **2005**, *123*, 204116.
4. Kirchner, B.; Spickermann, C.; Lehmann, S.B.; Perlt, E.; Langner, J.; von Domaros, M.; Reuther, P.; Uhlig, F.; Kohagen, M.; Brüssel, M. What can clusters tell us about the bulk?: Peacemaker: Extended quantum cluster equilibrium calculations. *Comput. Phys. Commun.* **2011**, *182*, 1428–1446.
5. von Domaros, M.; Perlt, E.; Ingenmey, J.; Marchelli, G.; Kirchner, B. Peacemaker2: Making clusters talk about binary mixtures and neat liquids. *SoftwareX* **2018**, *7*, 356–359.
6. Weinhold, F. Quantum cluster equilibrium theory of liquids: Illustrative application to water. *J. Chem. Phys.* **1998**, *109*, 373–384.
7. Haynes, W.M. *CRC Handbook of Chemistry and Physics*; CRC press: Boca Raton, Florida, 2014.
